# Urinary Fetuin-A Is a Novel Marker for Diabetic Nephropathy in Type 2 Diabetes Identified by Lectin Microarray

**DOI:** 10.1371/journal.pone.0077118

**Published:** 2013-10-15

**Authors:** Kentaro Inoue, Jun Wada, Jun Eguchi, Atsuko Nakatsuka, Sanae Teshigawara, Kazutoshi Murakami, Daisuke Ogawa, Takahiro Terami, Akihiro Katayama, Atsuhito Tone, Izumi Iseda, Kazuyuki Hida, Masao Yamada, Tomohisa Ogawa, Hirofumi Makino

**Affiliations:** 1 Department of Medicine and Clinical Science, Okayama University Graduate School of Medicine, Dentistry and Pharmaceutical Sciences, Kita-ku, Okayama, Japan; 2 Department of Diabetic Nephropathy, Okayama University Graduate School of Medicine, Dentistry and Pharmaceutical Sciences, Kita-ku, Okayama, Japan; 3 Department of General Medicine, Okayama University Graduate School of Medicine, Dentistry and Pharmaceutical Sciences, Kita-ku, Okayama, Japan; 4 National Hospital Organization Okayama Medical Center, Department of Diabetes and Metabolism, Kita-ku, Okayama, Japan; 5 GlycoTechnica Ltd., Aoba-ku, Yokohama, Japan; 6 GP BioSciences Co., Ltd., Aoba-ku, Yokohama, Japan; Hirosaki University Graduate School of Medicine, Japan

## Abstract

We analyzed the urine samples of patients with type 2 diabetes at various stages of diabetic nephropathy by lectin microarray to identify a biomarker to predict the progression of diabetic nephropathy. Japanese patients with type 2 diabetes at various stages of nephropathy were enrolled and we performed lectin microarray analyses (n = 17) and measured urinary excretion of fetuin-A (n = 85). The increased signals of urine samples were observed in Siaα2-6Gal/GalNAc-binding lectins (SNA, SSA, TJA-I) during the progression of diabetic nephropathy. We next isolated sialylated glycoproteins by using SSA-lectin affinity chromatography and identified fetuin-A by liquid chromatography–tandem mass spectrometer. Urinary excretion of fetuin-A significantly increased during the progression of albuminuria (A1, 0.40±0.43; A2, 0.60±0.53; A3 1.57±1.13 ng/gCr; p = 7.29×10^−8^) and of GFR stages (G1, 0.39±0.39; G2, 0.49±0.45; G3, 1.25±1.18; G4, 1.34±0.80 ng/gCr; p = 3.89×10^−4^). Multivariate logistic regression analysis was employed to assess fetuin-A as a risk for diabetic nephropathy with microalbuminuria or GFR<60 mL/min. Fetuin-A is demonstrated as a risk factor for both microalbuminuria and reduction of GFR in diabetic nephropathy with the odds ratio of 4.721 (1.881–11.844) and 3.739 (1.785–7.841), respectively. Collectively, the glycan profiling analysis is useful method to identify the urine biomarkers and fetuin-A is a candidate to predict the progression of diabetic nephropathy.

## Introduction

The most critical issue in clinical nephrology is relentless and progressive increase in the patients with end-stage renal disease (ESRD) in worldwide. The impact of diabetic nephropathy on the increasing population with chronic kidney disease (CKD) and ESRD is enormous. The intensified multifactorial intervention in patients with type 2 diabetes mellitus resulted in reduced risk of microangiopathy, cardiovascular events and mortality in Steno type 2 randomized studies [1]; however, the incidence of ESRD is progressively increasing in worldwide. To predict the progression of diabetic nephropathy and cardiovascular outcome, the simultaneous evaluation of albuminuria and glomerular filtration rate (GFR) is recommended by the KDIGO: Kidney Disease Improving Global Outcomes CKD Work Group [2]. In The Action in Diabetes and Vascular Disease: Preterax and Diamicron-MR Controlled Evaluation (ADVANCE) study, the measurements of albuminuria, eGFR or their combination predicted the cardiovascular events and death, and renal outcome [3]. In addition to the albuminuria at baseline, the changes of albuminuria further well-predicted mortality and cardiovascular and renal outcomes, independent of baseline albuminuria reported by ONTARGET investigators [4]. Although the repeated measurements of albuminuria is recommended in the clinical practice in diabetes, the presence of GFR decliners in both type 1 and type 2 diabetes has been reported. In type 1 diabetes, the GFR decliners with early reduction of GFR were reported in 9% of the patients with normoalbuminuria and 31% of microalbuminuria [5]. In the patients with type 2 diabetes, the rapid GFR decliners demonstrated the reduction of GFR although they were treated with olmesartan in addition to the angiotensin converting enzyme inhibitors. In such patients, it was difficult to predict the natural course of diabetic nephropathy by the combination of albuminuria and eGFR [6].

Based upon these clinical observations, we need to search more reliable urinary biomarkers to predict both renal and cardiovascular outcome. The biomarkers of renal dysfunction such as transferrin, type IV collagen and N-acetyl-β-D-glucosaminidase, inflammatory markers including orosomucoid, tumour necrosis factor-α, transforming growth factor-β, vascular endothelial growth factor and monocyte chemoattractant protein-1, as well as oxidative stress markers such as 8-hydroxy-2′deoxyguanosine may be more sensitive than urinary albumin, the current gold standard, in the detection of incipient nephropathy and risk assessment of cardiovascular disease; however, the sensitivity of these markers compared with albumin requires further investigation [7].

Recently, the urinary proteome analyses have been performed using 2-dementional gel electrophoresis and subsequent mass spectrometry to identify the novel urinary markers [8]–[10]; however, the identification of new markers may be suffered from contamination of urinary major proteins such as albumin, immunoglobulins, α1-antitrypsin, transferrin, and haptoglobin. In the line of considerations, we focused on the alterations of glycochains to identify useful urinary biomarkers. The changes in glycoproteome profile in the urine may be due to the alterations in the glycoprotein leakage into the urine by the damages of capillary selective permeability and also attributed to the high glucose-induced changes in the expression of the enzymes which are responsible to the glycochain modification. For example, increased hexosamine biosynthesis induced by high glucose conditions plays a key role in the development of insulin resistance in primary cultured adipocytes [11] and the increased flux through the hexosamine biosynthetic pathway and subsequent enhanced O-linked glycosylation (N-acetylglucosamine [O-GlcNAc]) of proteins have been implicated in insulin resistance in skeletal muscle [12]. However, the glycoproteome profile has not been well-investigated because of the technical obstacles. We employed the evanescent-field fluorescence-assisted lectin microarray: a new strategy for glycan profiling, which allows sensitive, real-time observation of multiple lectin-carbohydrate interactions under equilibrium conditions, to identify the changes in the functional glycans in a high-throughput manner [13]. We identified the increase in the biding activity to Siaα2-6-Gal/GalNAc in urine samples from the patients with diabetic nephropathy. We next identified fetuin-A, α1-microglobulin, and orosomucoid as sialylated glycoproteins and we found fetuin-A may be a useful urinary marker to predict the development of microalbuminuria and reduction of GFR in diabetic nephropathy.

## Materials and Methods

### Patients

Urine samples of Japanese healthy subjects without type 2 diabetes (n = 12) and Japanese patients with type 2 diabetes with various stages of normoalbuminuria (n = 7), microalbuminuria (n = 5) and macroalbuminuria (n = 5) were obtained and subjected to lectin microarray studies. Based on the lectin microarray studies, we identified sialylated glycoproteins, such as fetuin-A, α1-microglobulin, and orosomucoid as candidate markers for diabetic nephropathy and we newly recruited Japanese patients with type 2 diabetes (n = 85, 62.9±11.3 years) into this study. The patients with type 2 diabetes were treated with oral hypoglycemic agents (n = 48) and insulin treatment (n = 49). The patients with eGFR <15 ml/min/1.73 m^2^ or under dialysis were excluded from the current study. All recruited patients with type 2 diabetes agreed to perform lectin microarray of urine samples and measure urinary levels of fetuin-A, α1-microglobulin, and orosomucoid. The study was conducted in accordance with the ethical principle of the Declaration of Helsinki and approved by ethical committee of Okayama University Graduate School of Medicine, Dentistry and Pharmaceutical Sciences. We obtained written informed consent from each patient.

### Lectin Microarray

Fifty mL of urine samples were concentrated by Centricon at 5,000 *g* for 40 min and further by Microcon at 14,000 *g* for 70 min to the volume of 0.5 mL (Millipore, Billerica, MA). Ten µL of concentrated urine samples were applied to Multiple Affinity Removal Spin Cartridge for Human Serum (Agilent Technologies, Santa Clara, CA) to remove major serum proteins such as albumin, IgG, α1-antitrypsin, IgA, transferrin, and haptoglobin. Five hundred µl of the effluents dialyzed against PBS were applied to ULTRAFREE 0.5 BIOMAX-5k (Millipore) and concentrated to final volume of 50 µL. Protein concentration was measured with MicroBCA Protein Assay Kit (Thermo Scientific Pierce, Rockford, IL) and the final concentration was adjusted to 50 µg/mL, in which 20 µL was incubated with Cy3 at room temperature for 1 hour. Cy3-labeled samples were applied to gel filtration columns (Zeba Desalt Spin Columns 0.5 ml, Thermo Scientific Pierce) and the samples with 2, 1, 0.5, 0.25, 0.125, 0.063, 0.031 µg/mL were prepared with Probing Buffer and 100 µL/well of samples were applied to Lectin Array, LecChip (GP Biosciences, Tokyo, Japan) at 20°C for 15 hours. The lectin signals were measured with Glycostation™ Reader 1200 with exposure time (133 msec) and gain (85, 95, 105, and 115). Scanned images of 16 bit TIFF were analyzed with Array-Pro Analyzer (MEDIA CYBERNETICS, Rockville, MD) and GlycoStation Tools (GP Biosciences). The list of lectins is indicated in the **Table 1** and blood group A antigen (HPA) and group B antigen (EEL) were excluded from the analysis.

**Table 1:** A list of lectins of LecChip^TM^Ver.1 and the specificity.

Lectin No.	Lectin	Origin	Reported specificity
1	LTL	*Lotus tetragonolobus*	Fuc α1-3(Galβ1-4)GlcNAc, Fuc α1-2Galβ1-4GlcNAc
2	PSA	*Pisum sativum*	Fuc α1-6GlcNAc, α-D-Glc, α-D-Man
3	LCA	*Lens culinaris*	Fuc α1-6GlcNAc, α-D-Glc, α-D-Man
4	UEA-I	*Ulex europaeus*	Fuc α1-2Galβ1-4GlcNAc
5	AOL	*Aspergillus oryzae l-fucose-specific lectin*	Fuc α1-6GlcNAc (core fucose)
6	AAL	*Aleuria aurantia*	Fuc α1-6GlcNAc, Fuc α1-3(Galβ1-4)GlcNAc
7	MAL	*Maackia amurensis*	Siaα2-3Galβ1-4GlcNAc
8	SNA	*Sambucus nigra*	Siaα2-6Gal/GalNAc
9	SSA	*Sambucus sieboldiana*	Siaα2-6Gal/GalNAc
10	TJA-I	*Trichosanthes japonica*	Siaα2-6Gal/GalNAc
11	PHAL	*Phaseolus vulgaris*	tri/tetra-antennary complex-type N-glycan
12	ECA	*Erythrina cristagalli*	Galβ1-4GlcNAc
13	RCA120	*Ricinus communis*	Galβ1-4GlcNAc
14	PHAE	*Phaseolus vulgaris*	bi-antennary complex-type N-glycan with outer Gal and bisecting GlcNAc
15	DSA	*Datura stramonium*	(GlcNAc β1-4)n, Galβ1-4GlcNAc
16	GSL-II	*Griffonia simplicifolia*	agalactosylated tri/tetra antennary glycans, GlcNAc
17	NPA	*Narcissus pseudonarcissus*	High-Mannose, Man α1-6Man
18	ConA	*Canavalia ensiformis*	High-Mannose, Man α1-6(Manα1-3)Man
19	GNA	*Galanthus nivalis*	High-Mannose, Man α1-3Man
20	HHL	*Hippeastrum hybrid*	High-Mannose, Man α1-3Man, Man α1-6Man
21	ACG	*Agrocybe cylindracea*	Siaα2-3Galβ1-4GlcNAc
22	TxLCI	*Tulipa gesneriana*	Man α1-3(Manα1-6)Man, bi- and tri-antennary complex-type N-glycan, GalNAc
23	BPL	*Bauhinia purpurea alba*	Galβ1-3GalNAc, GalNAc
24	TJA-II	*Trichosanthes japonica*	Fuc α1-2Galβ1-> or GalNAc β1-> groups at their nonreducing terminals
25	EEL	*Euonymus europaeus*	blood group B antigen, Galα1-3Gal
26	ABA	*Agaricus bisporus*	Galβ1-3GalNAc
27	LEL	*Lycopersicon esculentum*	GlcNAc trimers/tetramers
28	STL	*Solanum tuberosum*	GlcNAc oligomers, oligosaccharide containing GlcNAc and MurNAc
29	UDA	*Urtica dioica*	GlcNAc β1-4GlcNAc, Mixture of Man5 to Man9
30	PWM	*Phytolacca americana*	(GlcNAc β1-4)n
31	Jacalin	*Artocarpus integrifolia*	Galβ1-3GalNAc, GalNAc
32	PNA	*Arachis hypogaea*	Galβ1-3GalNAc
33	WFA	*Wisteria floribunda*	GalNAc β1-4GlcNAc, Galβ1-3(-6)GalNAc
34	ACA	*Amaranthus caudatus*	Galβ1-3GalNAc
35	MPA	*Maclura pomifera*	Galβ1-3GalNAc, GalNAc
36	HPA	*Helix pomatia agglutinin*	α-linked terminal GalNAc
37	VVA	*Vicia villosa*	α-linked terminal GalNAc, GalNAc α1-3Gal
38	DBA	*Dolichos biflorus*	blood group A antigen, GalNAc α1-3GalNAc
39	SBA	*Glycine max*	α- or β-linked termincal GalNAc, GalNAcα1-3Gal
40	Calsepa	*Calystegia sepium*	Mannose, Maltose
41	PTL-I	*Psophocarpus tetragonolobus*	α-linked terminal GalNAc
42	MAH	*Maackia amurensis*	Siaα2-3Galβ1-3(Siaα2-6)GalNAc
43	WGA	*Triticum unlgaris*	chitin oligomers, Sia
44	GSL-I A4	*Griffonia simplicifolia Lectin I Isolectin A4*	α-linked GalNAc
45	GSL-I B4	*Griffonia simplicifolia Lectin I Isolectin B4*	α-linked Gal

These data were collected from lectin vendors and reports found by internet searches.

### Isolation of Sialylated Urinary Proteins in the Patients with Diabetic Nephropathy

Hundred mL of urine samples were concentrated by Centricon at 5,000 *g* for 40 min and further by Microcon at 14,000 *g* for 70 min to the volume of 1 mL. Affinity chromatography was performed using SSA-Agarose (Lectin-Agarose Set-III) and BioLogic LP system II (#731-8300X2, BIO-RAD, Hercules, CA). The SSA-Agarose column was equilibrated by 6.0 mL of PBS at the flow rate of 0.2 mL/min. The concentrated urine samples of 1.0 mL were applied to the sample loop and PBS was loaded at 0.1 mL/min for 10 min. The SSA-Agarose column was washed with PBS at 0.1 mL/min for 70 min. Five mL of the elution buffer (0.2 M lactose) was applied to sample loop and eluted with PBS at 0.1 mL/min for 60 min and further washed with PBS at 0.5 mL/min for 20 min. While eluting the sialylated glycoproteins, the fractions of 0.5 ml were collected every 5 min. The eluted samples were subjected to SDS-PAGE analysis and the proteins were identified by Liquid chromatography–tandem mass spectrometer (LC/MS-MS) analyses as follows.

Cysteine bonds of the eluted glycoproteins were reduced by 10 mM dithiothreitol (DTT) at 56°C for 1 hour and alkylated with 50 mM iodoacetamide (IAA) at room temperature for 45 min in the dark. They were enzymatically digested with 0.1 µg of sequencing grade trypsin at 30°C for overnight. The digested peptides were extracted once in 1% formic acid and subsequently twice in 5% formic acid and in 50% acetonitrite. Peptides were separated by nanoUPLC (nanoACQUITY UPLC, Waters, Milford, MA) and analyzed with Q-Tof micro (Waters). nanoUPLC was equipped with 5.0 µm Symmetry C18, 180 µmID×2 cm precolumn and 1.7 µm BEH 130 C18, 100 µmID×10 cm column. Mobile phase A was water with 0.1% formic acid whilst mobile phase B was 0.1% formic acid in acetonitrile. Using MassLynx 4.1 (Waters) the MS/MS raw data were transformed into peak lists (.pklfiles) and they were searched thorough NCBInr and Swiss-Prot by using Mascot (Matrix Science, Boston, MA).

### Blood Sampling and Assays

We measured overnight fasting serum levels of total cholesterol, low density lipoprotein (LDL) cholesterol, high density lipoprotein (HDL) cholesterol, triglycerides (L Type Wako Triglyceride H, Wako Chemical, Osaka, Japan), uric acid, creatinine (Cr), and urea nitrogen (UN). We also measured plasma glucose and HbA1c. Urinary albumin was measured in random spot urine samples by standard immuno-nephelometric assay. The urinary albumin-creatinine ratio (ACR) was calculated. Estimated glomerular filtration rate (eGFR) was calculated by equation; eGFR (ml/min/1.73 m^2^) = 194×Cr^−1.094^×age^−0.287^ in male and eGFR (ml/min/1.73 m^2^) = 194×Cr^−1.094^×age^−0.287^×0.739 in female [14]. By using the definition and classification of chronic kidney disease [Kidney Disease: Improving Global Outcomes (KDIGO)] [2], all patients were classified into albuminuria and GFR category. In albuminuria stages, the patients were classified into three groups; A1 (<30 mg/gCr), A2 (30–299 mg/gCr) and A3 (≥300 mg/gCr). In GFR stages, they were classified into 4 groups; G1 (>90 ml/min/1.73 m^2^), G2 (60–89 ml/min/1.73 m^2^), G3 (30–59 ml/min/1.73 m^2^), and G4 (15–29 ml/min/1.73 m^2^). Urinary excretions of fetuin-A, α1-microglobulin, and orosomucoid were measured with ELISA kit for Human Fetuin-A (BioVender, Modrice, Czech Republic), LZ Test Eiken α1-M (Eiken Chemical Co., Tokyo, Japan), and N Antiserum to Human α1-acid Glycoprotein (Siemens Healthcare Diagnostics Inc., Marburg, Germany).

### Statistical Analysis

All data are expressed as mean ± standard deviation (SD) values in tables. Urinary levels of fetuin-A, α1-microglobulin, and orosomucoid demonstrated non-normal distribution and medians with interquartile range were indicated in box plot in Figures. Spearman correlation coefficients were used to evaluate whether urinary levels of fetuin-A, α1-microglobulin, and orosomucoid correlated with various parameters. To determine the variables independently associated with urinary levels of fetuin-A, α1-microglobulin, and orosomucoid in the patients with type 2 diabetes, multiple regression analysis was performed by including estimated glomerular filtration rate (eGFR), albumin/creatinine ratio and HDL cholesterol (HDL-C) as independent variables. Urinary levels of fetuin-A, α1-microglobulin, orosomucoid and various clinical parameters in albuminuria and GFR stages were compared by Kruskal-Wallis test. Multivariate logistic regression analysis to access the urinary fetuin-A, α1-microglobulin, orosomucoid excretions as a risk for diabetic nephropathy with microalbuminuria or with GFR<60 mL/min. *P* values less than 0.05 were considered statistically significant. Statistical analysis was performed with IBM SPSS Statistics Base and IBM SPSS Regression (IBM, Armonk, NY).

## Results

### Lectin Microarray Analyses Demonstrated the Increased Binding Activity to Sia*α*2-6-Gal/GalNAc

**Figure 1:**
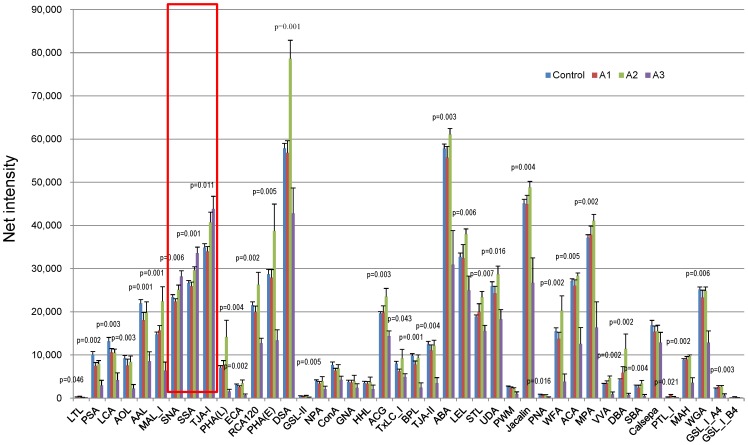
Lectin microarray analysis using urine samples from the patients with various albuminuria stages. Lectin microarray analysis of urine samples were performed in the healthy subjects without type 2 diabetes (Control, n = 12) and the patients with type 2 diabetes with various stages of normoalubuminuria (A1, n = 7), microalbuminuria (A2, n = 5) and macroalbuminuria (A3, n = 5). Signals to various lectins are compared by Kruskal-Wallis test.

We performed lectin microarray analyses and compared the urine samples of the healthy subjects without type 2 diabetes (n = 12) and the patients with type 2 diabetes with various stages of normoalubuminuria (n = 7), microalbuminuria (n = 5) and macroalbuminuria (n = 5). The reactivity to the many lectins, such as fucose binder (PSA, LCA, AOL, and AAL), Lac/LacNAc binder [PHA(L), ECA, RCA120, PHA(E)], α- or β-Gal binder (BPL, ABA, PNA, ACA), chitobiose binder (DSA, LEL, STL, UDA, PWM, WGA), and α- or β-GalNAc binder (Jacalin, WFA, MPA, VVA, DBA, SBA, PTL-I, GSL-IA4), significantly declined at the stage of macroalbuminuria (**Figure 1**). Among them, lectins which bind to N-glycosylation, RCA120, PHA(E), DSA, demonstrated the increased binding activity at the stage of microalbuminuria. Notably, in contrast to majority of the lectins, the binding to Siaα2-6-Gal/GalNAc (SNA, SSA, TJA-1) progressively increased in the albuminuria stages of diabetic nephropathy (**Figure 1** **, red box**). Since we identified specific increase in the biding activity to Siaα2-6-Gal/GalNAc in urine samples in the patients with diabetic nephropathy, we next screened the sialylated glycoproteins in the urine samples of diabetic nephropathy.

### Fetuin-A, *α*1-microglobulin and Orosomuioid were Identified by SSA-Agarose Column Chromatography and LC/MS-MS Analyses

**Figure 2:**
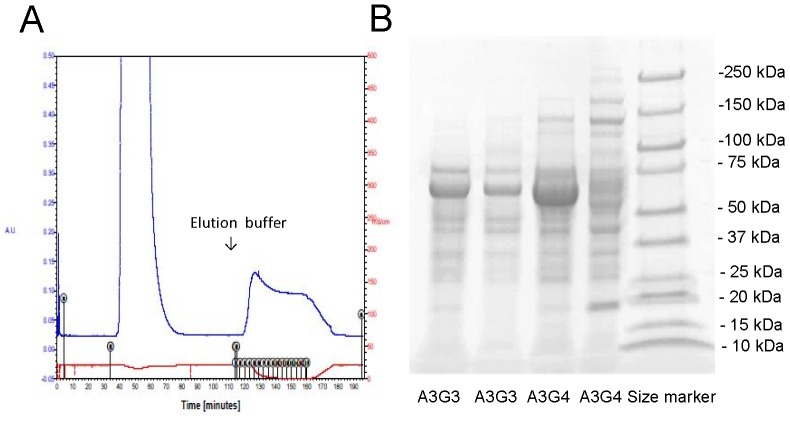
SSA-Agarose column choromatography performed in the 4 patients with type 2 diabetes. **A**. The concentrated urine samples were applied to SSA-Agarose column, washed with PBS and eluted with 0.2 M lactose. **B**. The effluents from the patients manifested with various albuminria and GFR stages, A3G3 and A3G4, were subjected to SDS-PAGE and stained with Coomassie Brilliant Blue. The bands were visualized and they were subjected to liquid chromatography-tandem mass spectrometer (LC/MS-MS) analysis.

SNA- and SSA-agarose were commercially available and we could isolate the glycoproteins by SSA-agarose in preliminary experiments. Thus, we performed SSA-Agarose column choromatography and the effluents were subjected to SDS-PAGE in **Figure 2**. We confirmed that the bands visualized with Coomassie Brilliant Blue staining increased in the patients with CKD stage of A3G4 compared with the patients A3G3. The effluents were subjected to LC/MS-MS and raw data of the proteins hit by Mascot program searching through NCBInr and Swiss-Prot database were indicated in **Tables 2** **and** **3** in the patients with CKD stages of A3G3 and A3G4, respectively. The listed proteins demonstrated the serum major proteins such as albumin, immunoglobulins, complements, α1-antitrypsin, transferrin, and haptoglobin. However, we identified three sialylated glycoprpteins such as α1-microglobulin (Protein AMBP), α1-acid glycoprotein (orosomucoid) and fetuin-A (α2-HS-glycorptein). Fetuin-A [15], α1-microglobulin [16], and orosomucoid [17] have been reported as sialylated glycoproteins and we further validated the significance of urinary excretion of sialylated glycoproteins as biomarkers for diabetic nephropathy.

**Table 2:** Liquid chromatography–tandem mass spectrometer (LC/MS-MS) of samples from the patients with A3G3 and the search result through NCBInr and Swiss-Prot database performed by Mascot.

Pos.	Ac. No.	Protein Name	Sequences	emPAI* ^1^	Score* ^2^
1	ALBU_HUMAN	Serum albumin	36	11.04	3985
2	TRFE_HUMAN	Serotransferrin	15	1.08	965
3	AMBP_HUMAN	Protein AMBP (alpha 1-microglobulin)	5	0.57	224
4	VTDB_HUMAN	Vitamin D-binding protein	3	0.14	130
5	HEMO_HUMAN	Hemopexin	3	0.23	112
6	PTGDS_HUMAN	Prostaglandin-H2 D-isomerase	1	0.18	75
7	IGKC_HUMAN	Ig kappa chain C region	1	0.34	70
8	HPT_HUMAN	Haptoglobin	3	0.17	63
9	DTX3L_HUMAN	E3 ubiquitin-protein ligase DTX3L	1	0.04	49
10	CLUS_HUMAN	Clusterin	1	0.07	39
11	SAP_HUMAN	Proactivator polypeptide	1	0.06	34
12	A1AT_HUMAN	Alpha-1-antitrypsin	2	0.08	33
13	AFAM_HUMAN	Afamin	2	0.05	32
14	FETUA_HUMAN	Alpha-2-HS-glycoprotein (Fetuin-A)	1	0.09	29
15	THRB_HUMAN	Prothrombin	1	0.05	25
16	TRPC4_HUMAN	Short transient receptor potential channel 4	1	0.03	20
17	RABE1_HUMAN	Q15276	2	0.04	19
18	MARK1_HUMAN	Serine/threonine-protein kinase MARK1	1	0.04	16

* ^1^emPAI (Exponentially Modified Protein Abundance Index) is calculated for the estimation of absolute protein amount as follow; *emPAI* = 10 ^Nobserved/Nobservable^−1.

* ^2^Probability Based Mowse Score. Ions score is −10*Log(P), where P is the probability that the observed match is a random event. Individual ions scores >16 indicate identity or extensive homology (p<0.05).

**Table 3:** Liquid chromatography–tandem mass spectrometer (LC/MS-MS) of samples from the patients with A3G4 and the search result through NCBInr and Swiss-Prot database performed by Mascot.

Pos.	Ac.No.	Protein Name	Sequences	emPAI* ^1^	Score* ^2^
1	ALBU_HUMAN	Serum albumin	52	21.13	3829
2	TRFE_HUMAN	Serotransferrin	23	1.61	800
3	HPT_HUMAN	Haptoglobin	17	3.1	683
4	IGHG1_HUMAN	Ig gamma-1 chain C region	10	2.56	601
5	IGHG2_HUMAN	Ig gamma-2 chain C region	8	0.99	227
6	IGKC_HUMAN	Ig kappa chain C region	6	4.73	516
7	IGHA1_HUMAN	Ig alpha-1 chain C region	10	1.54	422
8	A2MG_HUMAN	Alpha-2-macroglobulin	18	0.46	417
9	A1AT_HUMAN	Alpha-1-antitrypsin	10	1.16	392
10	APOA1_HUMAN	Apolipoprotein A-I	8	1.53	251
11	AMBP_HUMAN	Protein AMBP (alpha 1-microglobulin)	7	0.88	226
12	HEMO_HUMAN	Hemopexin	7	0.62	214
13	LAC2_HUMAN	Ig lambda-2 chain C regions	4	1.45	204
14	CO4A_HUMAN	Complement C4-A	2	0.04	147
15	CERU_HUMAN	Ceruloplasmin	2	0.06	127
16	IC1_HUMAN	Plasma protease C1 inhibitor	4	0.22	94
17	A1BG_HUMAN	Alpha-1B-glycoprotein	1	0.07	94
18	PTGDS_HUMAN	Prostaglandin-H2 D-isomerase	1	0.18	94
19	A1AG1_HUMAN	Alpha-1-acid glycoprotein 1 (orosomucoid)	3	0.56	82
20	ANGT_HUMAN	Angiotensinogen	1	0.07	74
21	ANT3_HUMAN	Antithrombin-III	2	0.07	72
22	KNG1_HUMAN	Kininogen-1	2	0.05	71
23	FETUA_HUMAN	Alpha-2-HS-glycoprotein (Fetuin-A)	1	0.09	70
24	PGRP2_HUMAN	N-acetylmuramoyl-L-alanine amidase	1	0.06	62
25	CO3_HUMAN	Complement C3	5	0.02	55
26	THRB_HUMAN	Prothrombin	1	0.05	31
27	VTDB_HUMAN	Vitamin D-binding protein	1	0.07	30
28	MTUS1_HUMAN	Microtubule-associated tumor suppressor 1	1	0.03	26

* ^1^emPAI (Exponentially Modified Protein Abundance Index) is calculated for the estimation of absolute protein amount as follow; *emPAI* = 10 ^Nobserved/Nobservable^−1.

* ^2^Probability Based Mowse Score. Ions score is −10*Log(P), where P is the probability that the observed match is a random event. Individual ions scores >16 indicate identity or extensive homology (p<0.05).

### Elevated Urinary Fetuin-A Excretion is a Risk for the Development of Diabetic Nephropathy

**Figure 3:**
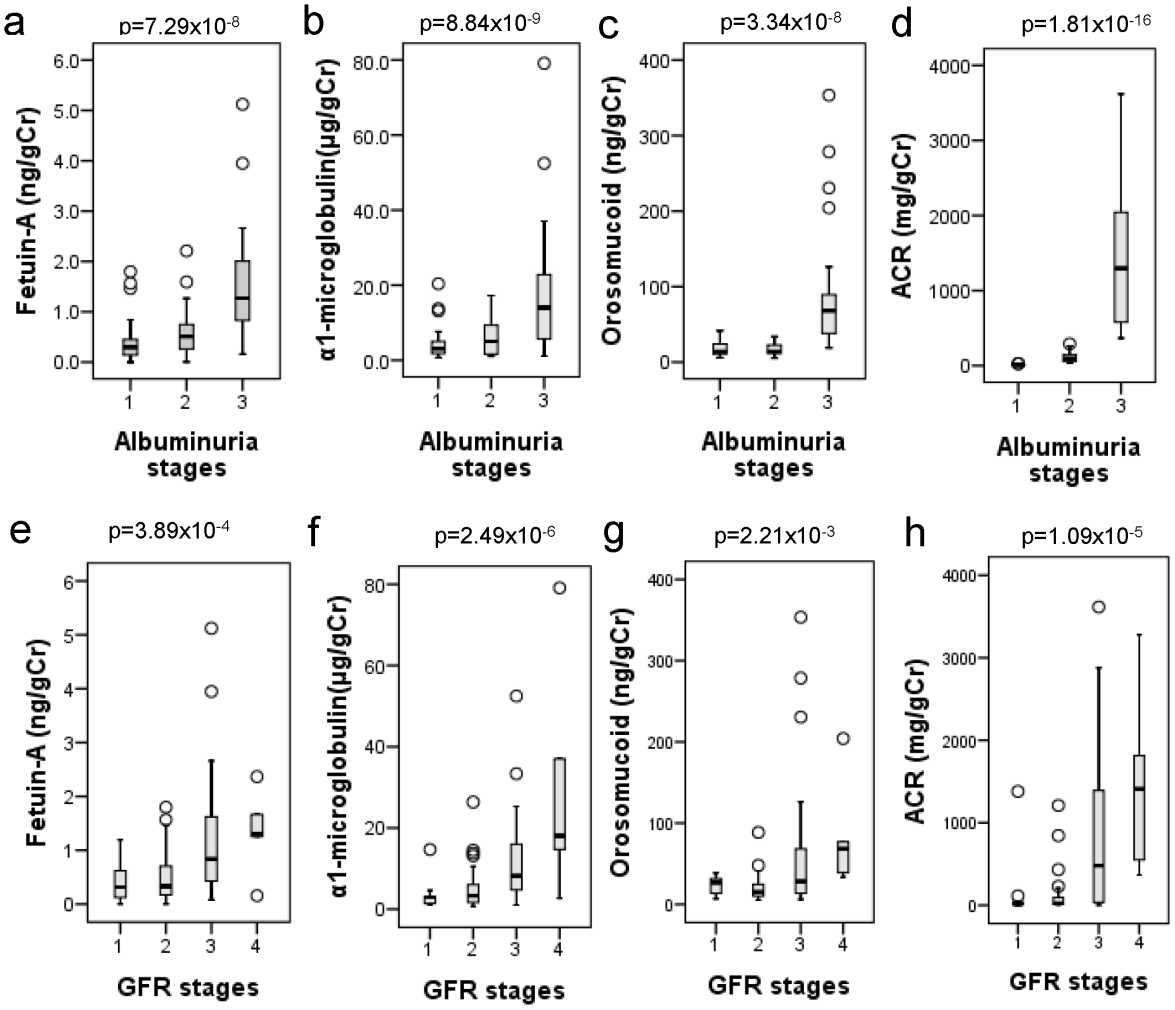
Urinary excretion of fetuin-A, α1-microglobulin, orosomucoid and albumin creatinine ratio (ACR) in various stages of diabetic nephropathy (n = 85). All of the urinary excretion of sialylated glycoprpteins such as fetuin-A, α1-microglobulin, and orosomucoid are compared by Kruskal-Wallis test.

We investigated urinary excretion of sialylated glycoproteins in various stages of diabetic nephropathy (n = 85). In albuminuria stages, age, serum total protein, serum albumin, Cr, UN, uric acid, HDL-C, eGFR, ACR were significantly changed revealed by Kruskal-Wallis test (**Table 4**). All of the urinary excretion of sialylated glycoprpteins such as fetuin-A, α1-microglobulin, and orosomucoid significantly increased during the progression of A1 to A3 stages (**Table 4** **and** **Figure 3a–d**). During the progression of GFR stages, serum total protein, serum albumin, Cr, UN, uric acid, eGFR, and ACR were significantly altered by Kruskal-Wallis test (**Table 5**). Like albuminuria stages, the urinary excretion of fetuin-A, α1-microglobulin, and orosomucoid significantly increased in the GFR stages from G1 to G4 revealed by Kruskal-Wallis test (**Table 5** **and** **Figure 3e–h**).

**Table 4:** Comparison of various parameters in albuminuria stages of chronic kidney disease in type 2 diabetes patients (n = 85).

	A1	A2	A3	Total	Kruskal-Wallis
Number (male/female)	36 (19/17)	25 (15/10)	24 (15/9)	85 (49/36)	
Age (years)	63.8±11.3	61.0±12.5	63.3±12.3	62.9±11.3	0.006*
BMI (kg/m^2^)	24.8±5.1	25.7±4.5	24.2±3.9	24.9±4.6	0.543
SBP (mmHg)	124.0±12.6	129.5±20.5	126.0±19.7	126.2±17.3	0.484
DBP (mmHg)	73.9±10.3	72.6±8.1	69.1±14.4	72.2±11.1	0.261
HbA1c (%)	7.31±0.64	7.24±0.90	7.38±1.17	7.31±0.87	0.850
Total protein (g/L)	70.4±4.3	70.7±4.8	66.1±6.5	69.3±5.4	0.003*
Albumin (g/L)	42.9±2.5	41.2±3.2	35.7±7.0	40.4±5.3	1.80×10^−16^ **
Cr (µmol/L)	66.4±13.3	78.3±26.3	144.2±70.3	91.9±52.3	4.86×10^−10^ **
UN (µmol/L)	5.5±1.5	7.1±2.7	10.0±3.8	7.3±3.3	5.92×10^−8^ **
Uric acid (µmol/L)	305.8±61.5	352.8±96.2	396.2±68.0	344.6±83.1	9.68×10^−5^ **
T-Cho (mmol/L)	5.09±0.94	4.86±0.84	5.06±1.14	4.99±0.97	0.689
TG (mmol/L)	1.65±0.92	1.70±1.10	2.16±1.74	1.81±1.26	0.780
HDL-C (mmol/L)	1.49±0.41	1.35±0.31	1.23±0.39	1.38±0.39	0.031*
LDL-C (mmol/L)	2.85±0.81	2.70±0.65	2.80±0.95	2.79±0.80	0.271
eGFR (mL/min)	74.5±16.3	67.9±19.2	42.4±19.0	63.5±22.4	6.66×10^−9^ **
ACR (mg/gCr)	12.7±6.0	114.3±72.6	1424±996	441.2±812	1.81×10^−16^ **
Fetuin-A (ng/gCr)	0.40±0.43	0.60±0.53	1.57±1.13	0.79±0.87	7.29×10^−8^ **
α1-microglobulin (µg/gCr)	4.24±4.03	6.30±5.12	17.83±18.08	8.68±11.74	8.84×10^−9^ **
Orosomucoid (ng/gCr)	17.5±9.1	17.9±8.7	91.4±87.2	38.5±57.0	3.34×10^−8^ **

BMI, body mass index; SBP, Systolic Blood Pressure; DPB, Diastolic Blood Pressure; Cr, serum creatinine; UN, serum urea nitrogen; T-Cho, Total cholesterol; TG, Triglyceride; HDL-C, HDL cholesterol; LDL-C, LDL cholesterol; eGFR, estimated glomerular filtration ratio; ACR, albumin/creatinine ratio;

* p<0.05;

** p<0.01.

**Table 5:** Comparison of various parameters in glomerular filtration stages of chronic kidney disease in type 2 diabetes patients (n = 85).

	G1	G2	G3	G4	Total	Kruskal-Wallis
Number (male/female)	9 (6/3)	42 (22/20)	29 (19/10)	5 (2/3)	85 (49/36)	
Age (years)	51.9±13.9	62.9±10.3	66.5±9.2	59.6±15.3	62.9±11.3	0.647
BMI (kg/m^2^)	27.6±8.1	25.1±4.6	24.1±3.1	22.6±2.2	24.9±4.6	0.155
SBP (mmHg)	129.7±13.8	127.6±17.3	124.0±19.1	120.2±11.1	126.2±17.3	0.640
DBP (mmHg)	76.5±13.8	74.6±8.0	69.5±12.4	59.4±9.2	72.2±11.1	0.006
HbA1c (%)	7.54±0.79	7.27±0.83	7.40±0.94	6.68±0.82	7.31±0.87	0.323
Total protein (g/L)	70.4±3.7	70.9±4.4	67.6±6.2	63.0±5.0	69.3±5.44	0.002*
Albumin (g/L)	41.4±4.6	42.3±2.9	38.3±6.6	33.4±5.0	40.4±5.3	1.10×10^−4^ **
Cr (µmol/L)	60.9±16.0	65.9±11.6	115.7±40.7	227.1±88.2	91.9±52.3	1.89×10^−17^ **
UN (µmol/L)	5.5±2.2	5.8±1.6	8.6±2.7	14.6±5.1	7.3±3.3	9.85×10^−12^ **
Uric acid (µmol/L)	329.1±39.5	312.8±74.4	388.7±82.6	391.4±99.8	344.6±83.1	6.59×10^−4^ **
T-Cho (mmol/L)	5.11±0.96	4.97±0.89	5.06±1.02	4.52±1.39	4.99±0.97	0.695
TG (mmol/L)	2.04±1.30	1.68±1.06	1.95±1.57	1.58±0.63	1.81±1.26	0.487
HDL-C (mmol/L)	1.25±0.26	1.44±0.37	1.32±0.41	1.45±0.51	1.38±0.39	0.427
LDL-C (mmol/L)	2.96±0.95	2.79±0.67	2.83±0.92	2.28±0.87	2.79±0.80	0.740
eGFR (mL/min)	96.2±15.6	74.8±8.1	44.4±9.2	20.6±8.3	63.5±22.4	1.16×10^−30^ **
ACR (mg/gCr)	179.7±451.6	108.3±227.7	824.5±0.80	1484±1168	441.2±812	1.09×10^−5^ **
Fetuin-A (ng/gCr)	0.39±0.39	0.49±0.45	1.25±1.18	1.34±0.80	0.79±0.87	3.89×10^−4^ **
α1-microglobulin (µg/gCr)	3.74±4.26	4.94±4.92	11.90±11.04	30.32±29.93	8.68±11.74	2.49×10^−6^ **
Orosomucoid (ng/gCr)	22.5±11.4	19.7±14.8	62.7±84.3	84.4±69.4	38.5±57.0	2.21×10^−3^ **

BMI, body mass index; SBP, Systolic Blood Pressure; DPB, Diastolic Blood Pressure; Cr, serum creatinine; UN, serum urea nitrogen; T-Cho, Total cholesterol; TG, Triglyceride; HDL-C, HDL cholesterol; LDL-C, LDL cholesterol; eGFR, estimated glomerular filtration ratio; ACR, albumin/creatinine ratio;

* p<0.05;

** p<0.01.

**Figure 4:**
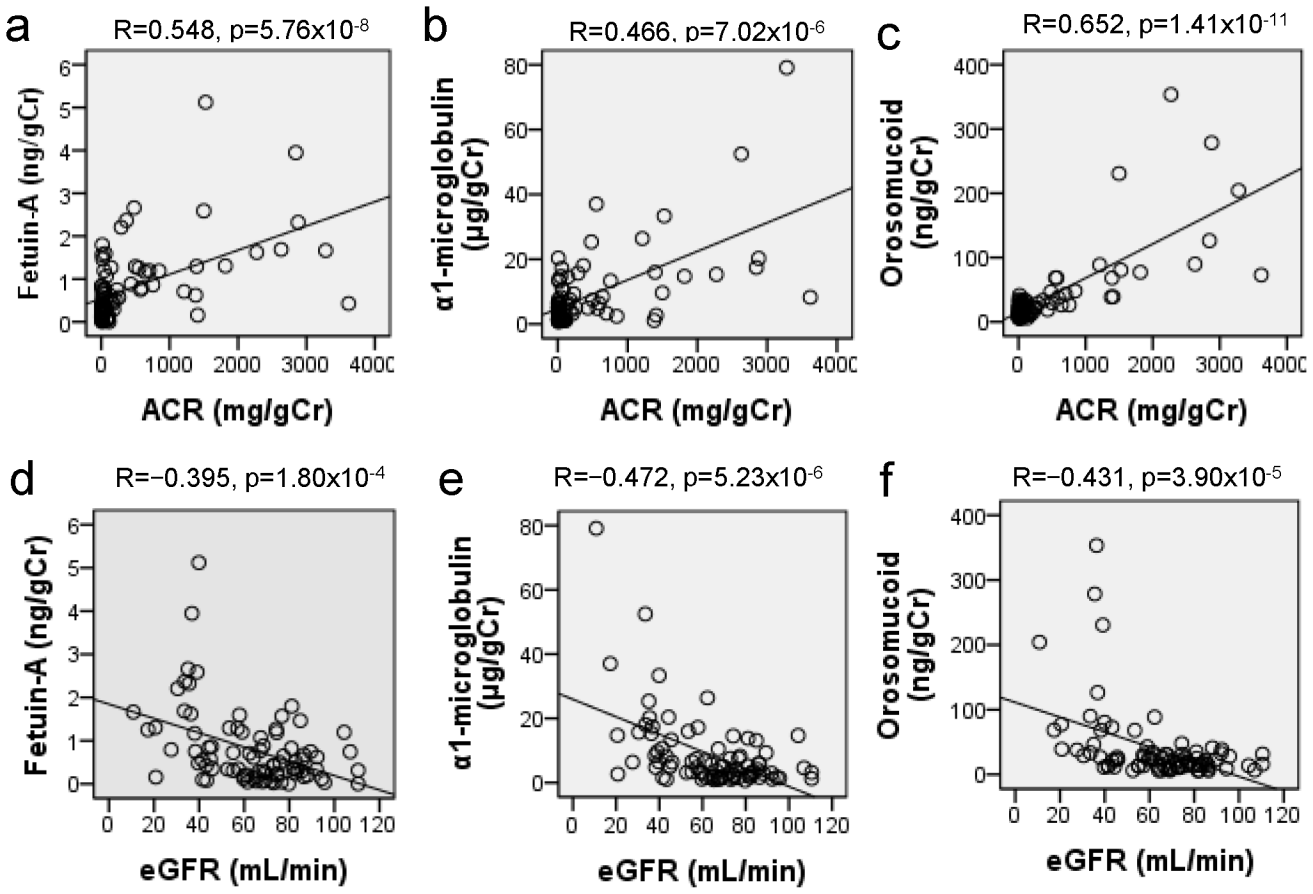
Simple correlation of urinary excretion of fetuin-A, α1-microglobulin, orosomucoid with estimated glomerular filtration ratio (eGFR) and urinary albumin creatinine ratio (ACR) in the patients with diabetic nephropathy (n = 85). Spearman correlation coefficients are used to evaluate whether urinary levels of fetuin-A, α1-microglobulin, and orosomucoid correlate with eGFR and ACR.

All of the urinary excretion of fetuin-A, α1-microglobulin, and orosomucoid positively correlated with Cr, UN and ACR and negatively correlated with serum albumin, HDL-C and eGFR with statistically significant differences (**Table 6** **and** **Figure 4**). The linear regression analyses were followed by a multiple regression analysis using the urinary excretion of fetuin-A, α1-microglobulin, and orosomucoid as the dependent variables to further analyze the significant predictors (**Table 6**). eGFR, ACR and HDL-C were used as independent variables. eGFR and ACR independently and significantly predicted urinary excretion of fetuin-A and α1-microglobulin. For urinary excretion of orosomucoid, ACR and HDL-C were significantly determinants in multiple regression models in **Table 7**. Finally, multivariate logistic regression analysis was employed to assess three urinary sialylated glycoproteins as a risk for diabetic nephropathy with microalbuminuria or GFR<60 mL/min. We used the forward stepwise method and the variable whose addition causes the largest statistically significant change in −2 Log Likelihood is added to the model. The final models are indicated in **Tables 8** and only fetuin-A was demonstrated as a risk factor for both microalbuminuria and reduction of GFR in diabetic nephropathy with the odds ratio (95% confidence intervals) of 4.721 (1.881–11.844) and 3.739 (1.785–7.841), respectively.

**Table 6:** Simple correlation of urinary sialylated glycoprotein excretions with various clinical parameters in the patients with type 2 diabetes (n = 85).

	Fetuin-A (ng/gCr)	α1-microglobulin (µg/gCr)	Orosomucoid (ng/gCr)
Age (years)	R = 0.009, p = 0.937	R = 0.123, p = 0.261	R = −0.008, p = 0.944
BMI (kg/m^2^)	R = −0.139, p = 0.205	R = −0.067, p = 0.541	R = −0.032, p = 0.770
SBP (mmHg)	R = 0.043, p = 0.693	R = −0.005, p = 0.964	R = 0.103, p = 0.348
DBP (mmHg)	R = −0.145, p = 0.186	R = −0.214, p = 0.049*	R = −0.027, p = 0.807
HbA1c (%)	R = 0.113, p = 0.307	R = 0.110, p = 0.318	R = 0.056, p = 0.612
Total protein (g/L)	R = −0.261, p = 0.017*	R = −0.275, p = 0.012*	R = −0.213, p = 0.053
Albumin (g/L)	R = −0.377, p = 4.36×10^−4^ **	R = −0.376, p = 4.67×10^−4^ **	R = −0.394, p = 2.28×10^−4^ **
Cr (µmol/L)	R = 0.368, p = 5.23×10^−4^ **	R = 0.388, p = 2.40×10^−4^ **	R = 0.399, p = 1.53×10^−4^ **
UN (µmol/L)	R = 0.405, p = 1.31×10^−4^ **	R = 0.439, p = 2.96×10^−5^ **	R = 0.363, p = 6.85×10^−4^ **
Uric acid (µmol/L)	R = 0.079, p = 0.474	R = 0.073, p = 0.509	R = 0.295, p = 0.006**
T-Cho (mmol/L)	R = −0.099, p = 0.372	R = −0.080, p = 0.471	R = 0.062, p = 0.576
TG (mmol/L)	R = 0.060, p = 0.582	R = 0.055, p = 0.615	R = 0.186, p = 0.088
HDL-C (mmol/L)	R = −0.313, p = 0.004**	R = −0.258, p = 0.017*	R = −0.244, p = 0.025*
LDL-C (mmol/L)	R = −0.007, p = 0.948	R = −0.043, p = 0.697	R = 0.067, p = 0.544
eGFR (mL/min)	R = −0.395, p = 1.80×10^−4^ **	R = −0.472, p = 5.23×10^−6^ **	R = −0.431, p = 3.90×10^−5^ **
ACR (mg/gCr)	R = 0.548, p = 5.76×10^−8^ **	R = 0.466, p = 7.02×10^−6^ **	R = 0.652, p = 1.40×10^−11^ **

BMI, body mass index; SBP, Systolic Blood Pressure; DPB, Diastolic Blood Pressure; Cr, serum creatinine; UN, serum urea nitrogen; T-Cho, Total cholesterol; TG, Triglyceride; HDL-C, HDL cholesterol; LDL-C, LDL cholesterol; eGFR, estimated glomerular filtration ratio; ACR, albumin/creatinine ratio;

* p<0.05;

** p<0.01.

**Table 7:** Multiple linear regression analysis using urinary sialylated glycoprotein excretions as dependent variables in the patients with type 2 diabetes (n = 85).

Dependent variable	Independent variable	Unstandardized coefficient		Standardized coefficient	t value	P value	Adjusted R^2^
		B	Standard Error	Beta			
Fetuin-A	eGFR (mL/min)	−0.076	0.042	−0.196	−1.813	0.074	0.335
(ng/gCr)	ACR (mg/gCr)	0.004	0.001	0.395	3.645	4.71×10^−4^ **	
	HDL-C (mmol/L)	−4.048	2.035	−0.182	−1.989	0.050	
α1-microglobulin	eGFR (mL/min)	−0.138	0.053	−0.263	−2.617	0.011*	0.423
(µg/gCr)	ACR (mg/gCr)	0.007	0.001	0.461	4.560	4.71×10^−5^	
	HDL-C (mmol/L)	−1.443	2.548	−0.048	−0.566	0.573	
Orosomucoid	eGFR (mL/min)	−0.136	0.212	−0.053	−0.642	0.523	0.605
(ng/gCr)	ACR (mg/gCr)	0.049	0.006	0.703	8.405	1.19×10^−12^ **	
	HDL-C (mmol/L)	−26.65	10.240	−0.183	−2.603	0.011	

Estimated glomerular filtration rate (eGFR), albumin/creatinine ratio and HDL cholesterol (HDL-C) are used as independent variables in stepwise multiple linear regression analysis in model 1. In model 2, all parameters are included in the analysis.

* p<0.05;

** p<0.01.

**Table 8:** Stepwise multivariate logistic regression analysis to assess the urinary sialylated glycoprotein excretions as a risk for diabetic nephropathy with microalbuminuria or glomerular filtration rate (GFR)<60 ml/min.

Risk factor for microalbuminuria	B	Standard error	p	Odds ratio (95% confidentintervals)	Predictive accuracy
Fetuin-A (ng/gCr) (1SD increments)	1.784	0.539	9.424×10^−4^ **	4.721 (1.881–11.844)	74.1%
Risk factor for GFR<60 mL/min	B	Standard error	p	Odds ratio (95% confident intervals)	Predictive accuracy
Fetuin-A (ng/gCr) (1SD increments)	1.516	0.434	4.755×10^−4^ **	3.739 (1.785–7.841)	72.9%

** p<0.01.

## Discussion

Glycans have important roles in living organisms with their structural diversity; however, glycan profiling studies have not been extensively performed because it is technically challenging. Recently, the genome-wide association study identified hepatocyte nuclear factor 1-α (HNF1A) as a key regulator of fucosylation and the DG9-glycan index, which is the ratio of fucosylated to nonfucosylated triantennary glycans, display altered fucosylation of N-linked glycans on plasma proteins. Thus, the glycan biomarkers could improve the efficiency of a diagnosis of HNF1A-MODY [18]. In diabetic nephropathy, Ahn J.M. *et al.* performed glycan profile of plasma samples from normal subjects and the patients with diabetes. They captured glycoproteins by multi-lectin affinity chromatography and trypsin-digested glycoproteins were subjected to the analysis by LC-MS/MS [19]. However, no other studies have been reported to survey the glycan profile of the urine samples so far, and we believe that the current investigation is the first study to perform glycan profiling of urines samples from the patients with diabetic nephropathy. As a result, we have found that global reduction of the bindings to lectins, such as fucose, Lac/LacNA, α- or β-Gal, chitobiose, and α- or β-GalNAc binders in urine samples of diabetic nephropathy at macroalbuminuria stage. Unlike the reduced bindings to these lectins, the biding activity to Siaα2-6-Gal/GalNAc binders progressively increased at micro- and macroalbuminruia stages. In the patients with type 1 diabetes, the reduction of sialidase activities was observed in mononuclear leucocytes and they speculated that diabetes-associated changes in sialylation of functional cell surface glycolconjugates may have important clinical consequences in diabetes [20]. The analysis of sialylation of insulin-like growth factor-binding protein (IGFBP)-3 from the poorly controlled patients with type 2 diabetes, increased binding of IGFBP-3 to SNA suggesting increased sialylation of IGFBP3 [21]. In contrast, reduced α2-6 sialylation of glycodelin-A was observed in gestational diabetes mellitus [22]. One can speculate that the alterations in the expression of sialyltransferases or sialidase may influence the sialylation of plasma glycoproteins; however, the status of sialylation seems to be complex in the patients with diabetes. Increased sialylated glycoproteins in urine samples may also be due to the alteration in the permselectivities of glomerular capillary, since sialylated glycoproteins are characterized by negative charge.

α1-microglobulin, also known as protein HC (for Heterogeneous Charge), was initially suggested as a marker for the detection of proximal tubular dysfunction by cadmium poisoning [23]. α1-microglobulin is a small protein with up to 31 kDa and it is filtered through glomeruli and reabsorbed by the proximal tubules [24]. Urinary excretion of α1-microglobulin was significantly higher in the early stages of the disease process, while albumin excretion was still in the normal range [25]. Thus, it may serves as early marker for tubular damages in diabetic nephropathy and may precede albumin excretion to the urine [25]–[27]. Low molecular weight markers of tubular dysfunction such as α1-microglobulin, therefore, appear as markers of renal dysfunction that may complement markers of glomerular dysfunction such as albumin [7]. Orosomucoid, α-1 acid glycoprotein with 41 kDa, is an acute-phase protein and the serum concentrations correlated with low-grade inflammation in the patients with diabetes [28], [29]. Urine excretion of orosomucoid was increased in the patients with diabetes and normoalbuminuria and it correlated with markers of inflammation such as CRP [29]–[31] and markers for endothelial dysfunction [32]. Type 2 diabetic patients with elevated urinary orosomucoid excretion exhibited normal glomerular and tubular function, suggesting the possibility of local renal production of orosomucoid due to chronic low-grade inflammation rather than hyperfiltration [33].

Fetuin-A has been principally studied as an inhibitor for ectopic calcium deposition in the renal field and it is also an important promoter for insulin resistance. Fetuin-A, a liver secretory glycoprotein with 64 kDa, has been shown that it acts as carrier of free fatty acids (FFAs) and they are the intrinsic ligands for Toll-like receptor 4 (TLR4), which induces adipose tissue inflammation and insulin resistance [34]. Fetuin-A binds to the residues of Leu100-Gly123 and Thr493-Thr516 of TLR4 through the terminal galactoside moiety [34]. Thus, FFA-Fetuin-A induced TLR4 activation is very important in the lipid-induced inflammation and insulin resistance and type 2 diabetes. In addition, fetuin-A and adiponectin mediate the crosstalk between adipose tissues, liver and kidney. Fetuin-A suppresses mRNA expression of adiponectin in cultured human adipocytes and treatment of wild-type mice with fetuin-A lowered serum adiponectin levels [35]. Higher fetuin-A and lower adiponectin levels may contribute the development of insulin resistance, diabetes and subsequent obesity-related CKD and diabetic nephropathy [36]. Serum concentration of fetuin-A in type 2 diabetes patients has been reported and they positively correlated with macrovascular late complications in high-risk type 2 diabetes patients, while no association with metabolic status or microvascular complications [37]. Recent study indicated that serum fetuin-A is lower in microalbuminuria patients compared with normo- and mascroalbuminuric patients [38]. In other studies, lower serum levels of fetuin-A are associated with peripheral arterial disease in patients with type 2 diabetes [39] and serum fetuin-A levels are negatively associated with atherosclerotic calcified plaques [40]. Thus, the significance of serum fetuin-A levels is controversial whether it is a good marker for diabetic micro- and macrovascular complications. Unfortunately, we failed to detect changes in binding activities to various lectins in the sera from the patients with various stages of diabetic nephropathy (data not shown), we did not get a chance to measure the serum levels of fetuin-A. However, we demonstrated that the urinary excretion of fetuin-A is a candidate for the biomarker to predict the progression of diabetic nephropathy. Although two previous published studies identified fetuin-A in urines samples of the patients with diabetic nephropathy, the quantifications were limited to inaccurate estimations by fluorescence 2-D differential in-gel electrophoresis [41] and capillary electrophoresis coupled to mass spectrometry [42]. In contrast to previous studies, we firstly used stable ELISA kit to quantify the urinary excretion of fetuin-A. Urinary concentration of fetuin-A may be depending on the production from the liver, alterations in permeability through glomerular basement membrane by capillary damages and changes in tubular reabsorption. Higher excretion of fetuin-A into urine has been reported to reflect the insulin resistance and inflammatory responses in obesity and type 2 diabetes [43] and it may reflect the increase in the serum levels of fetuin-A and alterations in the changes in the permeability of glomerular capillaries. Fetuin-A is reported to pass through the slit diaphragm and re-introduced to proximal tubular cells by megalin-mediated endocytosis [44]. Zhou et al. also reported that urinary exosomal fetuin-A was increased in the rats treated with cisplatin injection and in the ICU patients with acute kidney injury [45]. Thus, an alternative explanation for increased urinary fetuin-A excretion in diabetic nephropathy could be due to the tubular injury. In our study, multivariate regression analysis indicated that higher urinary fetuin-A excretion demonstrated a higher risk for the development of microalbuminuria and reduction of renal function and future cohort study is required to further confirm this notion.

## Conclusions

In summary, the glycan profiling studies using urine samples from the patients with diabetic nephropathy is useful to identify the new biomarkers to predict the progression of diabetic nephropathy. We demonstrated that global reduction of the bindings to lectins, such as fucose, Lac/LacNA, α- or β-Gal, chitobiose, and α- or β-GalNAc binders in urine samples of diabetic nephropathy at macroalbuminuria stage, and in contrast increased biding activity to Siaα2-6-Gal/GalNAc binders. Further, we identified three sialylated glycoprpteins such as α1-microglobulin (Protein AMBP), α1-acid glycoprotein (orosomucoid) and fetuin-A (α2-HS-glycorptein) by SSA-Agarose column choromatography and LC/MS-MS analysis. Finally, we have newly shown that higher urinary excretion of fetuin-A is a risk factor for both microalbuminuria and reduction of GFR in diabetic nephropathy.
